# Graphene Oxide (GO)-Blended Polysulfone (PSf) Ultrafiltration Membranes for Lead Ion Rejection

**DOI:** 10.3390/membranes8030077

**Published:** 2018-09-06

**Authors:** Harish Ravishankar, Jens Christy, Veeriah Jegatheesan

**Affiliations:** School of Engineering, RMIT University, Melbourne, VIC 3000, Australia; harish.ravishankar@rmit.edu.au (H.R.); Jens.Christy@coliban.com.au (J.C.)

**Keywords:** graphene-oxide (GO), 1-methyl-2-pyrrolidone (NMP), lead (Pb), cross-flow filtration, ultrafiltration (UF) membranes

## Abstract

Graphene oxide (GO) has been widely reported and used for treatment of heavy metals from different waste streams. Although their use as additives for membranes has greatly enhanced membrane properties, there is still a bottleneck in obtaining membranes with high heavy-metal rejection efficiencies while maintaining high flux, mechanical strength, and porosity. In the present study, different compositions of GO (0–1 wt %)-blended membranes were prepared using 1-methyl-2-pyrrolidone (NMP) as solvent and water with 5% ethanol as non-solvent, and studied for the rejection of the chosen model heavy-metal lead. The prepared membranes were characterized for hydrophilicity, membrane porosity, flux, permeability, pore-size, mechanical strength, and membrane morphology. From the results, it was inferred that membranes having maximum GO in their blend (1 wt %) showed better hydrophilicity (water contact angle 34.2°), porosity (82.2%), permeability (52.1 L/m^2^ h bar), and pure water flux (163.71 L/m^2^ h) at 3-bar pressure as opposed to other compositions. The pore sizes of the membranes ranged between 18 to 24 nm. Tensile strength tests showed the role of GO as a positive reinforcement on the mechanical properties of membranes through Young’s modulus (188.13 ± 15.36 MPa) for the membrane having 0.25 wt % GO composition. Environmental Scanning Electron Microscopy (ESEM) images displayed the dense top layer supported by a porous, finger-like structure, obtained from instantaneous de-mixing favored by NMP and GO. The observed reduction in flux of lead solution for GO-blended membranes was due to osmotic pressure build-up caused by the retained nitrate salt by GO on the retentate side of the membrane. A maximum rejection of 98% was achieved with 1 wt % GO membrane at 1-bar pressure with flux of 43.62 L/m^2^ h, which decreased to 94% at 3-bar pressure with flux of 142.95 L/m^2^ h. These results showed how the application of NMP as solvent and GO as an additive could facilitate in obtaining high-flux and high-rejection membranes.

## 1. Introduction

Depleted freshwater resources has led to increased attention being given to the recycling and reuse of water [1]. Population expansion has augmented demand for materials, products, and infrastructure. This, in turn, has increased water consumption [2] and placed tremendous stress on energy demand [3]. Water and energy are the two most important sectors that are closely interwound and which play an impactful role in our society. Without fresh water, modern energy production (hydroelectric power, fracking, petroleum refining, cooling requirements for thermal energy generation, and bio-fuel production) would be impossible [3]. Similarly, treatment of drinking and waste-water and their distribution to and from the treatment plants consumes energy [3]. It has been reported that a total of 4% of energy is consumed for treatment purposes in the U.S. [3].

Industrial wastewater is predominantly laden with heavy metals and other contaminants [4]. With regulations in place to limit the discharge of these metals, cost-effective and efficient treatment technologies are being developed to decrease the stress this places upon the environment [5]. Material interfaces play a critical role in addressing this issue. The interface between aqueous solutions and solid materials (such as membranes) determine the properties and performance of membranes [3]. Membrane processes are reported to be effective in treating wastewater contaminated with heavy metals [5,6]. However, the downside to this membrane technology is low water recovery, fouling issues and high energy consumption [7]. These issues were reported to have been overcome through preparing blended membranes with enhanced permeability, rejection, anti-fouling properties which in turn reduce the investment and operational costs [8,9]. Graphene-oxide is one such common additive that is used for preparing blended membranes [10,11,12]. Graphene oxide blended polysulfone (PSf) composite membranes have been extensively characterized in different works [7,13,14]. It was confirmed that the addition of graphene oxide to the membranes displayed better porosity and prevented potential bacterial growth which was an issue with most filtration membranes [15]. Other beneficial attributes to using these blended membranes included better flux, enhanced hydrophilicity and improved mechanical properties [14,15].

The removal of different heavy metal ions using a commercial nanofiltration membrane (NF270) and its properties was reported by Al-Rashdi et al. [16]. This study looked at the effect of important parameters such as pH, pressure and metal concentration on the heavy metal rejection. The commercial membrane was reported to be successful in treating copper, cadmium, manganese and lead with rejections of 100%, 99%, 89% and 74% at pH = 1.50 and 4-bar pressure for an initial concentration of 1000 mg/L of heavy metal. Donnan repulsion and adsorption mechanism was reported to play a role in rejection. Heavy metal removal using GO blended membranes have been reported in literature. Recently, in a study [7] the application of graphene-oxide (GO) blended membranes for rejection of arsenate from water was reported to be successful. Dead end filtration was employed in this study and arsenate rejection of 83.65% was observed at pH 8.5 ± 0.2 and 4-bar pressure for a low initial concentration of 300 ± 10 μg/L. The predominant rejection mechanism was reported to be Donnan repulsion. Ganesh et al. [14] reported GO blended PSf membranes to be effective in removing high concentrations (1000 mg/L) of NaCl and Na_2_SO_4_ salts. The membrane blends having higher GO concentrations (2000 mg/L) were reported to show maximum rejections of 72% and 57% for Na_2_SO_4_ and NaCl salt. This membrane was proposed to be effective for desalination processes.

Mukherjee et al. [13] studied the effect of GO blended PSf membrane on adsorption of different heavy metal ions such as Cr (VI), Pb (II), Cu (II) and Cd (II). The study reported that the membrane showed high adsorption capacities for Pb^2+^ (79 mg/g), Cu^2+^ (75 mg/g), Cd^2+^ (68 mg/g) and Cr^6+^ (154 mg/g) at natural pH, 6.7, 6.5, 6.4 and 3.5, respectively. The membrane also showed a rejection between 90% and 96% for all the heavy metals mentioned at various operating conditions. Membrane blend having 0.2 wt % of GO was found to be optimum due to interplay of adsorption, diffusion and convection processes for heavy metal removal.

The above-mentioned studies on heavy metal rejection [13,14] employed dimethylformamide (DMF) as the solvent for membrane preparation. Madaeni and Rahimpour [17] reported on the effect of solvent on the performance of polyethersulfone (PES) and polysulfone (PSf) membranes. This study compared three different solvents namely *N*,*N*-dimethylacetamide (DMAc), *N*,*N*-dimethylformamide (DMF) and 1-methyl-2-pyrrolidone (NMP). From the comparative study it was found that the polysulfone membranes prepared using solvent NMP had higher porosity and flux as compared to membranes prepared using DMF. However, the polysulfone (PSf) membranes prepared using DMF showed better protein rejection as opposed to NMP. Application of GO blended PSf membranes prepared using NMP as solvent for heavy metal ion removal would be apt approach to improve rejection while maintaining the high porosity, flux and performance of the membrane.

In the present study we have focused on employing the advantages of GO by preparing blended membranes using NMP as solvent to treat lead present in water. Lead is a common heavy metal which is present in divalent form. Its contamination in water is widespread due to its release into the environment through industrial activities such as mining, battery manufacturing, ammunition, tetraethyl lead manufacturing, painting, dying and release through pipes [18]. Lead poisoning can cause severe damage to kidney, brain and reproductive system in humans [19]. Therefore, the World Health Organisation (WHO) has set the drinking water limit for lead as 0.01 mg/L [20].

Different GO blended PSf membranes with NMP as solvent were prepared using non-solvent induced phase separation process. The membranes were systematically characterised in terms of hydrophilicity, porosity, flux, pore size, mechanical properties and morphology. The characterised membranes were studied for their treatment ability to remove lead ions present in water. The effect of different concentrations of GO on lead removal was analysed to examine the treatment applicability of such membranes.

## 2. Materials and Methods

### 2.1. Materials

Polysulfone (PSf) (average Molecular Weight ~35,000) was purchased from Sigma Aldrich (Product Number: 428302), Sydney, NSW, Australia. Graphene oxide (GO) powder was purchased from Graphenea Inc., Boston, MA, USA. 1-methyl-2-pyrrolidone (NMP) (purity > 99.5%) was purchased from Merck, Sydney, NSW, Australia. Lead (II) nitrate was purchased from Sigma Aldrich (Product Number: 203580).

### 2.2. Preparation of Membrane

#### 2.2.1. Preparation of Casting Solution

PSf was dried in an oven overnight at 100 °C to get rid of moisture. It was placed in a desiccator the next morning to cool to room temperature, following which the required amount of PSf was added into the media bottle containing NMP at a defined concentration (refer to Table 1 for the composition) and stirred at 300 rpm at a temperature of 50 °C overnight. For GO-blended mixtures, required amounts of GO were added to NMP and sonicated for 1–3 h prior to the addition of PSf. After overnight mixing of the solution (with/without GO), sonication was performed for at least 1 h to ensure no air-bubbles were present in the solution mixture.

#### 2.2.2. Casting of the Membrane

Membranes were casted by pouring the casting solution onto the glass plate and using a casting knife (Elcometer, 643580, Elcometer Ltd., Manchester, UK) to manually draw the solution at constant speed to obtain a thin layer of 200 μm, at uniform thickness. After casting, the glass plate was immersed into a coagulation bath, having de-ionized water with 5% ethanol maintained at 20–30 °C to enable the de-mixing and phase inversion process to happen. After 10 min, the cured membrane was transferred to a fresh tray of de-ionized water for storage.

### 2.3. Characterisation

#### 2.3.1. Hydrophilicity—Water Contact Angle Measurement

To determine the hydrophilicity of the membranes, the static water contact angle was measured using a water contact angle goniometer (OCA20, Particle and surface science Pty. Ltd., Gosford, NSW, Australia). A water droplet was placed onto the membrane, and the contact angle was recorded at 90 s. Multiple readings were taken to try and obtain accurate results, and average values were reported.

#### 2.3.2. Membrane Porosity—Dry Wet Weight Method

Membrane porosity was calculated using the dry-wet weight method. 5 cm by 5 cm membrane samples were cut out and soaked overnight in water. The wet weight was measured using an analytical balance. The membrane samples were then dried at room temperature overnight and weighed the following day to obtain the dry weight. All weights were recorded multiple times on an analytical balance, and the thickness was measured using a digital Vernier caliper twice on each side of the samples. The values were then used in the following equation to calculate the porosity.

(1) $$\;Pr=\frac{M_{s}-M_{d}}{\rho AT}\;\;$$

where Pr = porosity; $M_{s}$ = saturated membrane weight; $M_{d}$ = dry membrane weight; ρ = density of water; A = Surface area of membrane; T = thickness of membrane.

#### 2.3.3. Membrane Permeation Test—Flux and Permeability Measurement

Membrane permeation tests were conducted to calculate the water flux of the membranes. The flux was determined by running deionized water and lead solution through the membranes at varying pressures (1, 2, and 3 bars) for 30 min through a cross-flow filtration unit. A membrane area of 0.0075 m^2^ was used for the tests conducted. The values, that is, the flow rate obtained was substituted in the equation below to calculate the flux:

(2) $$\;J_{v}=\frac{Q}{A}\;\;$$

where:

***J_v_***  permeate flux (L/m^2^ h)
***A***  effective membrane area (m^2^)
***Q***  volume flow rate (L/h)

The permeability of the membrane was calculated using the equation below.

(3) $$\;L_{p}=\frac{J_{v}}{\Delta P}\;\;$$

where:

***J_v_***  permeate flux (L/m^2^ h)
**Δ*P***  change in pressure (bar)
***L_p_***  water permeability (L/(m^2^ h bar))

#### 2.3.4. Mean Pore Size—Guerout-Elford-Ferry Equation

The porosity values obtained, as well as other parameters such as flow rate, membrane thickness, membrane surface area, and operational pressure, were used to calculate the pore size of the prepared membranes. The average pore size was estimated using the Guerout-Elford-Ferry equation, given below:

(4) $$\;r_{m}=\;\sqrt{\frac{\left(2.9-1.75\varepsilon \right)\times 8\eta lQ}{\varepsilon \;\times A\;\times \;\Delta P}}\;\;$$

where ***r_m_*** is the mean pore radius (m), ***η*** is the water viscosity, ***l*** is the membrane thickness (m), ***Q*** is the volume of the permeate water per unit time (m^3^/s), ***A*** is the effective area of the membrane (m^2^), ***ɛ*** is the porosity, and ***ΔP*** is the operational pressure (Pa).

#### 2.3.5. Mechanical Properties—Tensile Strength

The mechanical property of the prepared membranes was analyzed through tensile strength tests. The Instron 4467 tensile testing instrument (Model number: 4467, Instron, Bayswater, VIC, Australia) was used to determine the elongation point before break and Young’s modulus. Membrane samples of 15 mm wide and 100 mm long were cut and placed in between the clamps of the instrument. The thicknesses of the samples were measured using a digital thickness gauge and fed into the operating software (Bluehill version 1.9, Instron corporation, Canton, MA, USA). Tests were run at a strain rate of 2 mm/min and the values were recorded on the software. The elongation point before break and Young’s modulus were calculated based on five tests for each sample.

#### 2.3.6. Morphology—Environmental Scanning Electron Microscopy (ESEM)

The cross-section and surface morphology of the membranes were observed using Environmental Scanning Electron Microscopy (ESEM) (FEI Quanta 200, RMIT University, VIC, Australia). Thin samples were prepared and adhered on an aluminium stub before being coated with platinum (3 nm) using the precision etching and coating system (PECS). Samples were then analyzed at an operating voltage of 30 kV under both high and low vacuum operating conditions.

### 2.4. Cross-Flow Filtration Cell Setup and Lead Rejection Experiments

**Figure 1:**
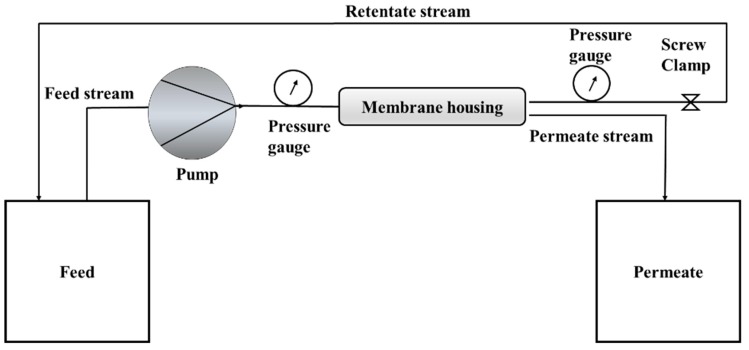
Schematic diagram of the cross-flow filtration cell.

A cross-flow filtration cell was used to evaluate the rejection of lead from the water. The filtration cell was made of stainless-steel membrane housing. Membranes with a surface area of 0.0075 m^2^ was cut out and placed in the housing. The retentate stream was sent back to the feed stream, thereby concentrating it. The feed flow pump frequency was set at 30 Hz. This was maintained constant during the experiment. The schematic of the cross-flow filtration cell is shown in Figure 1.

Lead solution of 50 mg/L was prepared from stock lead nitrate of 1000 mg/L. Lead rejection experiments were performed at three different pressures (1, 2, and 3 bars) at 20 °C. The pH of the solution was maintained at 6, using NaOH and HNO_3_. The permeate samples were collected after 30 min and analyzed for lead concentration by Atomic Absorption spectroscopy (AAS) Varian AA140 (Varian, Springvale, VIC, Australia) at 283 nm. The rejection was calculated using the following equation:

(5) $$\;R=1-\;\frac{C_{p}}{C_{f}}\;\;$$

where ***R*** is the rejection, and ***C_p_*** and ***C_f_*** are the concentration of lead in permeate and feed, respectively.

## 3. Results and Discussion

### 3.1. Characterisation of the Membranes

#### 3.1.1. Hydrophilicity—Water Contact Angle

**Figure 2:**
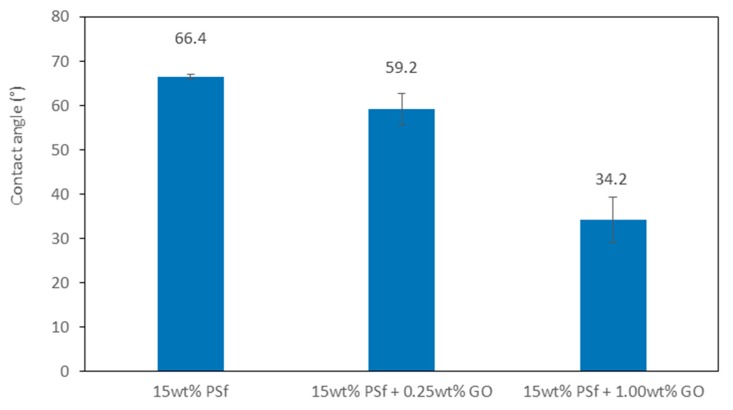
Water contact angle of different membranes.

The water contact angle or hydrophilicity reflects the wettability of membranes. When determining the hydrophilicity of the membranes, it was important to ensure the membranes were kept moist. Membranes with graphene oxide displayed a lower water contact angle (higher hydrophilicity), as compared to the pristine PSf membrane. Water contact was found to be lowest (34.2°) for the membrane blend with 1 wt % GO. The results demonstrate that the addition of GO improved the hydrophilic nature of the membranes. With the pure PSf membranes, the contact angle did not vary significantly, even after 5 min. This further confirmed the hydrophobic nature of PSf. Polysulfone is a high-strength material with negligible interaction capability with metal ions—hence, the addition of GO with its carboxyl, hydroxyl, and carbonyl groups enhances its ability to interact with metal ions as ion exchange and complexation is enhanced [21]. Figure 2 shows the water contact angles of the prepared membranes.

#### 3.1.2. Membrane Porosity and Pore Size

**Figure 3:**
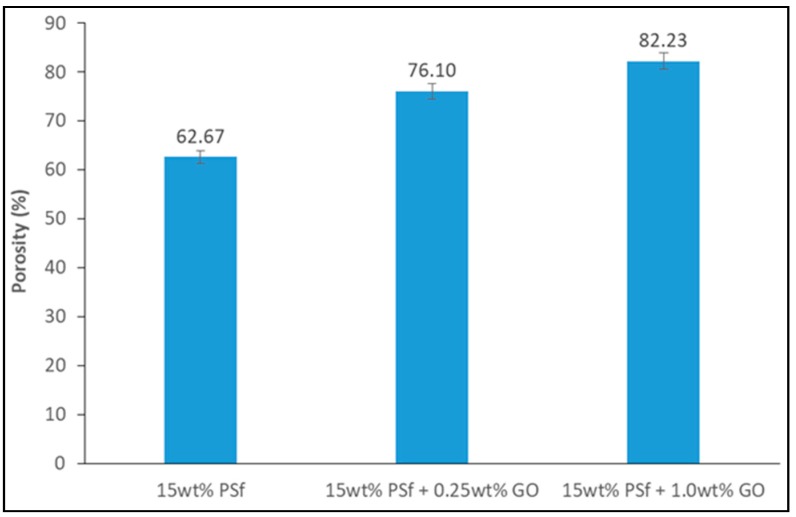
Porosity of the prepared membranes.

The membranes’ porosity was calculated using the dry-wet weight method. The porosity was observed to increase with an increase in GO concentration (Figure 3). The hydrophilicity effect of GO expedited the exchange of solvent/non-solvent during the phase inversion process, which led to higher membrane porosity. A similar observation was reported by Wang et al. [15] with PVDF membranes being blended with GO.

The prepared membranes had pore sizes ranging from 18 to 24 nm, confirming that the membranes were of ultrafiltration grade. The pore sizes of individual membranes are listed in Table 2. The addition of 0.25 wt % GO increased the pore size, and upon a further increment of GO to 1 wt %, the pore size decreased a bit but was not significant, as compared to the 0.25 wt % GO membrane.

#### 3.1.3. Membrane Permeation—Pure Water Flux and Permeability

**Figure 4:**
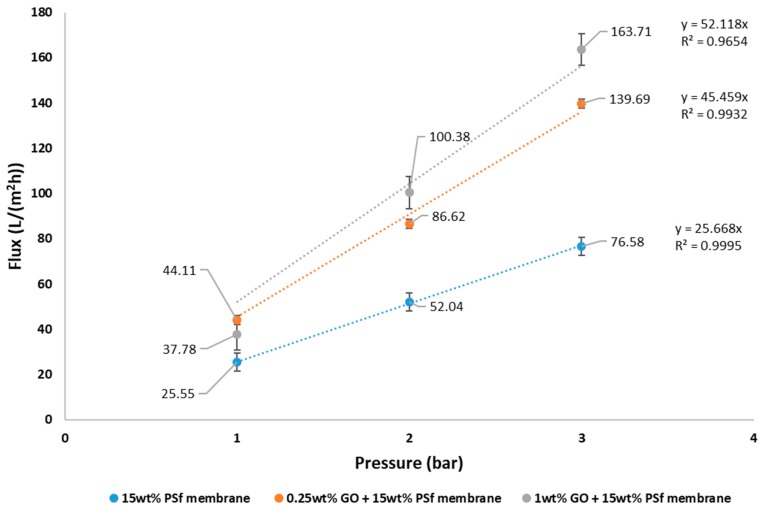
Pure water flux and permeability of prepared membranes.

The pure water flux of the prepared membranes at different pressures is shown in Figure 4. It can be observed from the graph that the flux increased with GO concentration in the membrane. The membrane having 1 wt % GO had a maximum flux of 163.71 L/m^2^ h at 3 bars. At 1 bar, 1 wt % GO had a lower flux than the 0.25 wt % GO. This may be due to the higher concentration of GO constricting the pores. Increase in flux with GO concentrations for PVDF ultrafiltration membranes have also been reported by Wang et al. [15]. The permeability of the membranes was also observed to increase with the addition of GO. The permeability was estimated at 25.7, 45.5, and 52.1 L/m^2^ h bar for 15 wt % PSf, 15 wt % PSf–0.25 wt % GO, and 15 wt % PSf–1 wt % GO, respectively (Figure 4.). The increase in permeability was due to the presence of GO favoring the solvent and non-solvent de-mixing.

**Figure 5:**
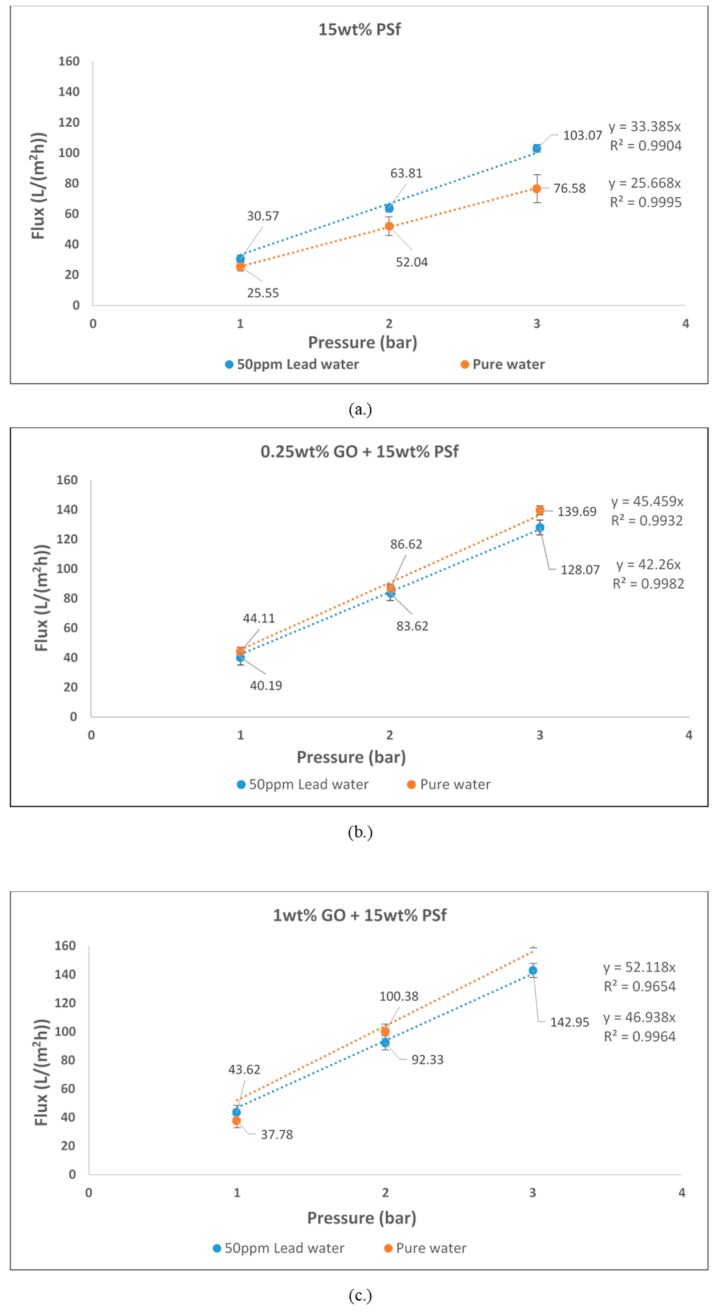
Pure water flux and 50 mg/L lead flux of (**a**) 15 wt % polysulfone (PSf); (**b**) 0.25 wt % graphene oxide (GO) and 15 wt % PSf; and (**c**) 1 wt % GO and 15 wt % PSf.

Figure 5 shows the fluxes of water and lead nitrate solution of different membranes. From the graphs it can be observed that the flux did increase linearly with pressure. This indicates that concentration polarisation did not occur as there was no significant reduction or dip in the flux achieved. However, graphene oxide (GO)-blended membranes did have lower fluxes for lead solution as opposed to pure water. This could have possibly been due to osmotic pressure build-up caused by the retained nitrate salt by GO on the retentate side of the membrane [16]. Although the fluxes reduced, it was still greater than the fluxes achieved for the pure PSf membrane. However, for pure PSf membranes, the flux for lead solution was higher compared to pure water flux. This can be attributed to the opening of the pores and absence of GO that could have retained the lead ions.

#### 3.1.4. Mechanical Properties—Tensile Strength

The mechanical properties of the prepared membranes were evaluated using the tensile strength test. From the tests, the tensile stress, Young’s modulus, and elongation at break was estimated (Table 2). It can be seen from Table 2 that the 0.25 wt % GO membrane was able to handle more tensile stress before permanent deformation occurred, which was then followed by the 15 wt % PSf membrane and then the 1.00 wt % GO membrane. It could be assumed that a small concentration perhaps did enhance the stress placed upon the polysulfone membrane—however, a higher concentration had an adverse effect on the membrane, thus causing it to fail under less stress. Young’s modulus also significantly increased for the membrane with 0.25 wt % GO, but decreased for the membrane with 1 wt % GO. The improvement in the membrane with 0.25 wt % GO concentration could be explained by the positive reinforcement brought by GO over the PSf crystallinity [22]. However, at 1 wt % GO, the mechanical properties were lost due to poor dispersion and formation of clusters throughout the membrane. In regard to the elongation of the membranes, the PSf had a shorter elongation length at break than the membranes with GO. However, the increment in elongation length was not substantial among the three membranes compared.

#### 3.1.5. Morphology of Membranes

**Figure 6:**
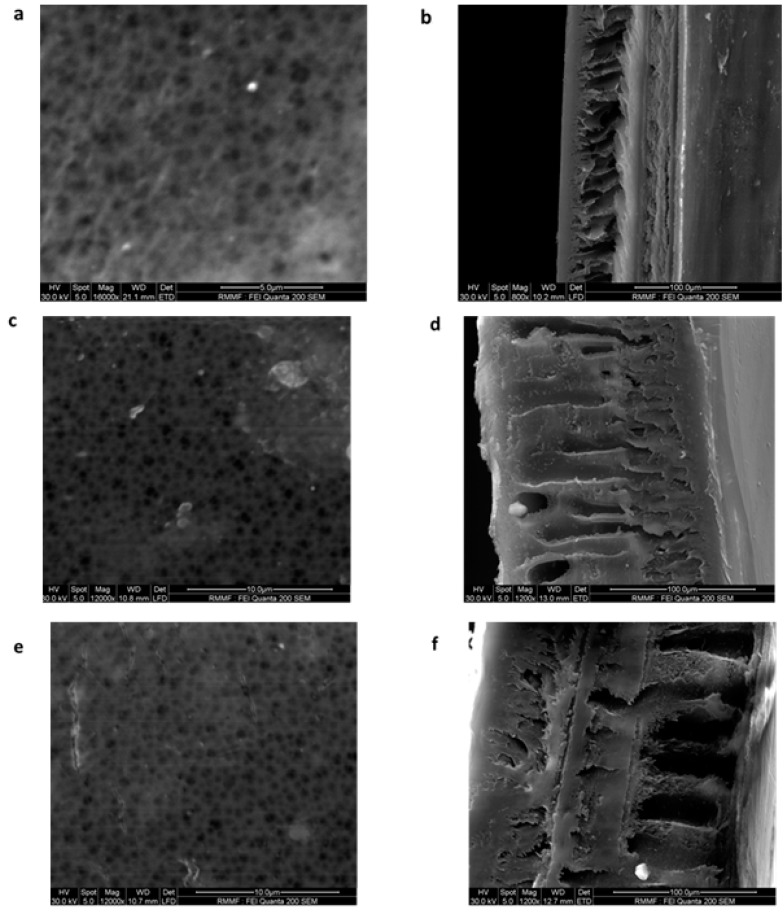
Environmental Scanning Electron Microscopy (ESEM) images of surface ((**a**)—mag 16,000×, (**c**)—mag 12,000× and (**e**)—mag 12,000×) and cross sections ((**b**)—800×, (**d**)—1200× and (**f**)—1200×) of membranes having compositions 15 wt % PSf (**a**,**b**), 15 wt % PSf–0.25 wt % GO (**c**,**d**), and 15 wt % PSf–1 wt % GO (**e**,**f**), respectively.

The surface and cross-sectional morphology of the membranes are shown in Figure 6. The ESEM of the surface images show the pores present on the membranes. The cross-sectional images of the membranes show the asymmetric structure, which is consistent with other studies that focused on morphology [17]. A dense top layer, which was supported by a porous, finger-like layer, was observed for all the membranes. The porous layer varied significantly with the addition of graphene oxide, from tighter and void-free pores to big macro-voids observed for the 1 wt % GO membrane (Figure 6f). The addition of GO increased the porosity of the membrane as it increased the macro-voids and reduced the thickness of the walls, thus increasing the permeability of the membrane. It was also noted that the hydrophilic nature of GO helped to form larger, longer pores and macro-voids [7].

Madaeni and Rahimpour [17] undertook a study using three different solvents, namely *N*,*N*-dimethylacetamide (DMAc), *N*,*N*-dimethylformamide (DMF), and 1-methyl-2-pyrrolidone (NMP) to study their effect on the morphology of polysulfone membranes. This study reported that membranes prepared using *N*,*N*-dimethylacetamide (DMAc) had higher porosity (89%) as compared to 1-methyl-2-pyrrolidone (NMP) and *N*,*N*-dimethylformamide (DMF), which were 83% and 80% respectively. The use of DMF resulted in a sponge-like membrane structure, which led to lower porosity. The difference in porosities were attributed to the solvent and non-solvent miscibility, where the lower the miscibility parameter (miscibility between solvent and non-solvent is high), the easier the movement of non-solvent through polymer and hence quicker de-mixing. The instantaneous de-mixing resulted in the porous top layer and finger-like pores in the supporting layer. This was reported on membranes that were formed using *N*,*N*-dimethylacetamide (DMAc) and 1-methyl-2-pyrrolidone (NMP) [16]. In our study, the use of NMP as solvent and water with 5% ethanol as non-solvent favored instantaneous de-mixing, thereby resulting in an asymmetrical porous top layer and a finger-like support layer (Figure 6b,d,e) and pore sizes ranging between 18–24 nm. The effect of 5% ethanol in water (non-solvent composition) resulted in membranes with smaller pore sizes (ultrafiltration range) as compared to an earlier study conducted by our research team [23] with water as a non-solvent, which resulted in membranes with pore sizes in the range of 175–250 nm (microfiltration range). This can be attributed to the higher miscibility parameter for the non-solvent (the presence of ethanol in water).

### 3.2. Lead Rejection

**Figure 7:**
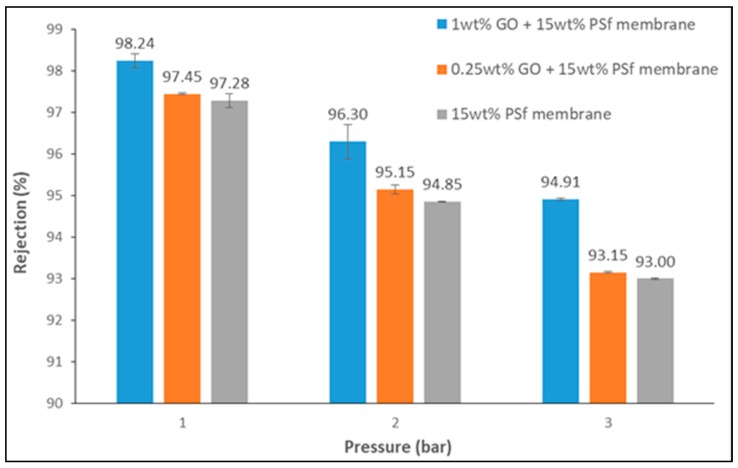
Lead rejection of the membranes at different pressures (lead concentration = 50 mg/L and pH = 5.5).

Rejection of ions can predominantly be achieved through adsorption, size exclusion, and charge exclusion [16]. The present study employed the use of GO to reject lead ions by employing the charge exclusion principle. Figure 7 shows the rejection of lead ions at different operating pressures by the different membranes prepared.

From the figure, it can be noted that higher rejection was achieved with the 1 wt % GO membrane at 1 bar of pressure. All membranes demonstrated a decrease in rejection when there was an increase in pressure, which can be attributed to the increase in water permeation which facilitated the convection of lead ions through the membrane pores.

A maximum lead rejection of 98% was achieved using a 1 wt % GO membrane operated at a pressure of 1 bar. This rejection decreased to 94% at 3 bars. Pristine PSf membrane showed a very high rejection rate of 93% at 3 bars. This high rejection by the pristine PSf membrane may have been due to the presence of smaller pores. The influence of GO could be seen with the increase in rejection, which was not very high but definite.

In a related study on heavy-metal removal by Mukerjee et al. [13] using GO-blended polysulfone membranes, a lead rejection rate of 95.5% was reported at pH 6.7 at 690 kPa for 50 mg/L of initial concentration for a 0.2 wt % GO membrane. The permeate flux of this membrane was reported to be around 32 L/m^2^ h at 414 kPa. In another study by Rezaee et al. [7], GO-blended PSf membrane (composition GO 1 wt % and 15 wt % PSf) was reported to have a maximum flux of 43.05 L/m^2^ h at 4-bar pressure, and showed 82% arsenic rejection. Ganesh et al. [14] also reported the GO-blended PSf membrane (25 wt % PSf and 1000 & 2000 mg/L GO) for Na_2_SO_4_ and NaCl rejection. From this study, it was observed that the solute rejection increased with GO doping. The membrane with the highest GO loading showed a maximum rejection of 72% for a concentration of 1000 mg/L of Na_2_SO_4_ at 4-bar pressure. However, this membrane showed a low water flux of 10 L/m^2^ h at 4 bar pressure. The three studies mentioned on salt rejection using GO-blended PSf membranes were prepared using DMF as a solvent. The usage of DMF was reported to change the membrane morphology (sponge-like structure) and thus reduce flux, compared to NMP on PSf membranes [17]. This study also highlighted better retention of protein by PSf membranes prepared using DMF as a solvent, as opposed to NMP and DMAc. This was attributed to the sponge-like structure that resulted in lower porosity and flux and highest retention.

In the present study, the GO-blended membrane with NMP as a solvent enabled the preparation membranes with high flux, porosity, and rejection performance. Lead rejection of 98% was achieved for the 1 wt % GO membrane at 1-bar pressure with a flux of 43.62 L/m^2^ h, and 94% at 3-bar pressure with a flux of 142.95 L/m^2^ h. The NMP facilitated a porous top layer and finger-like support structure for the membrane, which thus increased the membrane’s porosity and created flux. The addition of GO created a negative charge on the membrane that adsorbed the positively charged lead ions and thereby rejected them.

### 3.3. Performance of Membranes

The performance of the membrane is an important criterion that determines its applicability for real-time treatment. Membrane fouling is a major concern that deteriorates its performance. Fouling can be detected through an increase in transmembrane pressure (TMP) over time. The fouling effects and flux of GO-blended membranes were studied in detail in our previous research [23]. In that study, GO-blended membranes were used in a Membrane Bioreactor (MBR) system for treatment of lead-contaminated water. The results from the study showed that GO-blended membranes tended to foul slower than pristine PSf membranes. TMP profiles of 15 wt % PSf and 15 wt % PSf + 1 wt % GO showed that the PSf membrane was able to reach 55 kPa in around 60 days, and the GO-blended PSf membrane could reach a maximum of 30 kPa during the same period, while also taking 85 days to reach 55 kPa. The positive effects of GO on filtration cycle time, cleaning frequency, and anti-fouling were demonstrated in that study [23]. With decreased cleaning frequency, the membrane operation and their reusability can be extended over a longer period, thereby making membrane operation economically feasible. Operating membranes below their critical flux would minimize fouling and maximize the life of membranes.

## 4. Conclusions

GO-blended PSf ultrafiltration membranes were successfully prepared using NMP as a solvent. The prepared membranes displayed enhanced hydrophilicity and porosity, which improved their overall flux and permeability as compared to the GO-free membrane. The pore size of ultrafiltration membranes ranged from 18 nm to 24 nm. The addition of 0.25 wt % GO to PSf improved Young’s modulus, which, however, decreased at 1 wt % GO concentration due to poor dispersion and formation of clusters in the membrane. The positive impact of GO incorporation was observed with increased elongation at break lengths for GO-blended membranes. ESEM micrographs revealed the presence of a porous top layer and finger-like support layer in the prepared membranes. The prepared membranes had enhanced flux and permeability, and lead-rejection experiments showed that 1 wt % GO-blended PSf membranes had the highest rejection rate of 98% at 1-bar pressure, which, however, decreased with an increase in pressure and with a decrease in GO concentration. The results from this study highlight the role of GO and NMP in obtaining a highly porous membrane that provides improved flux and enhanced rejection of lead ions without compromising on membrane performance.

## Figures and Tables

**Table 1:** Composition of prepared membranes.

Sample	GO (wt/wt, %)	PSf (wt/wt, %)	NMP (wt/wt, %)	Total (g)
1	0.00	15%	85.00	100
2	0.25	15%	84.75	100
3	1.00	15%	84.00	100

**Table 2:** Characterization of prepared membranes.

Membrane	Young’s Modulus (MPa) (Mean ± 3 S.D.)	Tensile Stress (MPa) (Mean ± 3 S.D.)	Elongation at Break (mm) (Mean ± 3 S.D.)	Pore Size (nm) (Mean ± 3 S.D.)
15 wt % PSf	130.73 ± 15.84	3.54 ± 0.23	10.23 ± 4.36	17.36 ± 5.4
15 wt % PSf–0.25 wt % GO	188.13 ± 15.36	6.31 ± 0.47	14.83 ± 3.75	23.72 ± 6.9
15 wt % PSf–1 wt % GO	79.46 ± 28.57	1.10 ± 0.83	12.48 ± 6.5	22.73 ± 9.6

Note: S.D.—standard deviation.
